# Interaction of cytochrome P450 3A4 with cannabinoids and the drug darifenacin

**DOI:** 10.1016/j.jbc.2025.110709

**Published:** 2025-09-11

**Authors:** Irina F. Sevrioukova

**Affiliations:** Department of Molecular Biology and Biochemistry, University of California, Irvine, California, USA

**Keywords:** cannabinoid, cytochrome P450, CYP3A4, crystal structure, darifenacin, spectroscopy, structure–function, substrate binding

## Abstract

Cytochrome P450 3A4 (CYP3A4) is an important drug-metabolizing enzyme whose substrate binding mechanism remains incompletely understood because of insufficient structural information. This study investigated how CYP3A4 interacts with cannabinoids, (−)-*trans*-Δ^9^-tetrahydrocannabinol (THC), cannabidiol and cannabinol, and a muscarinic receptor blocker, darifenacin, using spectral, mutagenesis, and structural approaches. It was found that THC and cannabidiol act as type I ligands and induce a nearly complete high-spin transition in CYP3A4 (*K*_*d*_ of 1.9 μM and 3.6 μM, respectively), whereas cannabinol causes only negligible spectral changes. In the crystal structure, THC approaches the heme with the cyclohexenyl C7 and C8 atoms, the main sites of metabolism, without triggering any significant structural perturbations. Darifenacin is also a type I ligand but has two binding sites (*K*_*d*_ of 11 μM and 712 μM) and associates to the high-affinity site in the crystal structure, where it adopts an arched conformation, placing the dihydrobenzofuran moiety above the heme suitably for the ring opening and C7 hydroxylation, the main routes of metabolism. Polar interactions with S119 and R212 facilitate but are not essential for the THC binding, likely driven by hydrophobic interactions and steric complementarity with the active site. In contrast, H-bonding to S119 is critical for the complex formation with darifenacin. The new X-ray models have expanded the structural library of productive complexes of CYP3A4 and helped identify a mechanism through which local changes in the active site could transmit to the remote areas to further optimize substrate binding and promote metabolism.

Cytochrome P450 3A4 (CYP3A4) is a major human drug-metabolizing enzyme abundantly expressed in the liver and small intestine, where it catalyzes a variety of mono-oxygenation reactions, including hydroxylation, epoxidation, and heteroatom dealkylation. The extreme substrate promiscuity of CYP3A4 stems from its large and malleable active site that can accommodate diverse substrates differing in size and chemical structure (1). Besides endobiotics, such as steroid hormones, arachidonic acid, and bile acids, CYP3A4 biotransforms over half of administered medications, converting them into more water-soluble forms to facilitate excretion. Through degradation of drugs, CYP3A4 can lower their bioavailability and therapeutic efficiency, and, *vice versa*, drug plasma levels can be increased if CYP3A4 is inhibited. Moreover, the ability of CYP3A4 to simultaneously bind multiple substrates could lead to clinically relevant drug–drug interactions.

Mechanistic, allosteric, and structural aspects of CYP3A4–ligand interaction have been extensively investigated (2, 3, 4, 5, 6, 7, 8, 9, 10). The accumulated structural information was critical for understanding the ligand-binding mechanism and provided a foundation for computational studies and modeling techniques currently used for *in silico* identification of protein–ligand contacts and sites of metabolism (11, 12, 13, 14). However, the predictability of CYP3A4–ligand interactions remains poor, largely because of the high flexibility and adaptability of CYP3A4 to a wide range of substrates and a limited number of available substrate-bound structures. To date, 11 cocrystal structures of CYP3A4 with substrates have been reported (15, 16, 17, 18, 19, 20, 21, 22), only five of which represent productive complexes. Here, we report spectral and mutagenesis data on the interaction of CYP3A4 with cannabinoids, (−)-*trans*-Δ^9^-tetrahydrocannabinol (THC), cannabinol (CBN), and cannabidiol (CBD), and a muscarinic receptor blocker, darifenacin. In addition, we determined crystal structures of productive complexes with THC and darifenacin, which helped better understand the ligand-binding process and conformational dynamics of CYP3A4.

## Results and discussion

### Interaction of CYP3A4 with cannabinoids

#### Complex formation with THC

**Figure 1:**
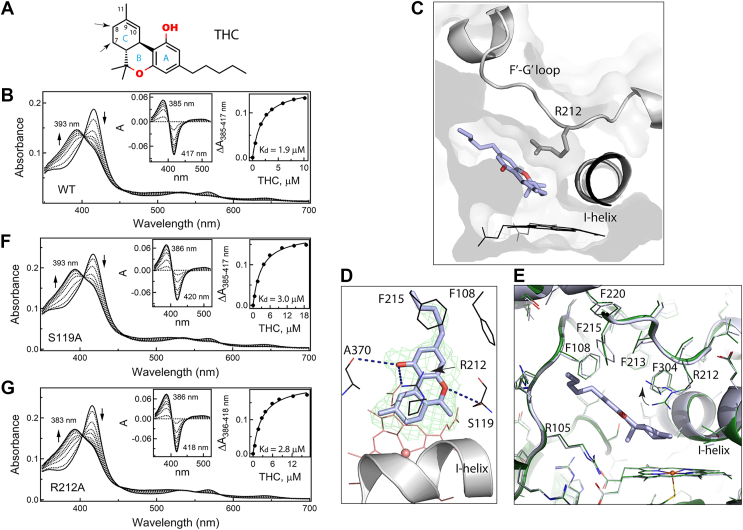
**Interaction of CYP3A4 with THC.***A,* chemical structure of THC. The tricyclic core consists of benzene, pyran, and cyclohexenyl rings (rings *A*–*C*, respectively). The sites preferably oxidized by CYP3A4 are indicated by *arrows*. *B,* spectral changes observed during equilibrium titration of WT CYP3A4 with THC. Direction of absorbance changes is indicated by *arrows*. *Left and right insets* are the difference spectra and titration plot with hyperbolic fitting, respectively. The derived *K*_*d*_ value is indicated. *C,* slice through the CYP3A4 molecule with a semitransparent surface to show the relative orientation of THC in the active site. *D,* a magnified *top view* at the active site. Long-range polar interactions of THC with the S119 and R212 side chains and the A370 carbonyl (4.2–4.5 Å distance) are shown as *black dotted lines*. *Green mesh* is a polder omit electron density map contoured at the 3σ level. *E,* superposition of THC-bound (in *pale blue*) and ligand-free CYP3A4 (in *green*; 5VCC structure) showing subtle adjustments in F108, F213, F215, and F220 and a rotameric switch in the R212 side chain to avoid steric clashing and optimize interactions with THC. *F* and *G,* spectral changes observed during equilibrium titration of the S119A and R212A mutants of CYP3A4, respectively, with THC. Direction of absorbance changes is indicated by *arrows*. *Left and right insets* show the difference spectra and titration plots with hyperbolic fittings, respectively. The derived *K*_*d*_ values are indicated. CYP3A4, cytochrome P450 3A4; THC, (−)-trans-Δ9-tetrahydrocannabinol.

Cannabinoids are extensively metabolized by hepatic CYPs, with over 40 metabolites of THC identified in humans (23). Earlier research suggested that 11-OH-THC is the major metabolite primarily produced by CYP2C9 (24), whereas CYP3A4 is responsible for the formation of 8-OH-THC and 9,10-epoxy-THC (25). However, a recent thorough and comprehensive study (26) that used modern mass spectrometry analysis for identification of THC-metabolizing CYPs and their products showed that at physiological THC concentrations (2–10 μM), CYP3A4 forms 8-OH- and 7-OH-THC (Fig. 1*A*), whereas the epoxy metabolite could not be detected. This suggests that 9,10-epoxy-THC may be produced only at high THC concentrations (64–130 μM) (25, 27).

Our spectral titrations showed that THC is a type I ligand that induces a blue shift in the Soret band of CYP3A4, leading to a nearly complete low-to-high spin transition (Fig. 1*B*). Hyperbolic fitting to the titration plot was consistent with a single-site THC binding, giving the dissociation constant (*K*_*d*_) of 1.9 ± 0.1 μM. Thus, THC is a strong binder, and its cocrystallization with CYP3A4 was straightforward.

#### Crystal structure of the CYP3A4–THC complex

The X-ray structure was solved to 2.72 Å resolution (Table 1) and contains one THC molecule bound to the active site. THC approaches the heme with the cyclohexenyl ring (C-ring) of the tricyclic core at a 40° incline angle, whereas the aliphatic tail points toward the substrate channel (Fig. 1*C*). No significant structural reorganization is required to accommodate THC. The RMSD between the C_α_ atoms of the THC-bound and ligand-free CYP3A4 (5VCC structure) is only 0.228 Å. One slight adjustment is observed in the I-helix, where two central residues, F304 and A305, shift away by 0.41 to 0.46 Å to allow THC to approach the catalytic center. As seen from Figure 1*E*, THC induces subtle changes in the tilt and rotational angles of F108, F213, F215, and F220 as well. These residues are part of the Phe-cluster, a dynamic structural element specific for CYP3A4 that could influence the ligand binding by changing the shape/volume of the active site. The most notable change is in the R212 side chain, whose guanidine group moves up and orients parallel to the tricyclic moiety of THC, establishing cation–π interactions with the partially overlapping benzene (A-ring). The ligand-binding mode is further stabilized by multiple van der Waals contacts as well as long-range polar interactions formed by the A-ring hydroxyl group with the A370 carbonyl and R212 guanidine and by the pyran (B-ring) oxygen with the S119 hydroxyl (4.2–4.5 Å distance; Fig. 1*D*). The aliphatic tail is flexible and not well defined in the crystal structure. However, its hydrophobic interactions with the neighboring F108 and F215 could contribute to the binding affinity of THC.

**Table 1:** Data collection and refinement statistics

Ligand/ Protein Data Bank code	THC (9PLJ)	Darifenacin (9PLK)
Data statistics		
Space group	I222	I222
Unit cell parameters	*a* = 76 Å, *b* = 102 Å, *c* = 125 Å	*a* = 76 Å, *b* = 101 Å, *c* = 123 Å
	α, β, γ = 90°	α, β, γ = 90°
Resolution range (Å)	79.18–2.72 (2.87–2.72)^a^	78.34–2.25 (2.37–2.25)
Total reflections	70,004 (8139)	108,133 (16,296)
Unique reflections	13,315 (1875)	22,740 (3288)
Redundancy	5.3 (4.3)	4.8 (5.0)
Completeness	98.7 (97.3)	99.2 (99.6)
Average *I*/*σI*	7.6 (1.0)	10.1 (1.3)
*R*_merge_	0.120 (1.936)	0.063 (1.665)
*R*_pim_	0.055 (0.978)	0.032 (0.822)
CC ½	0.996 (0.377)	0.999 (0.372)
Refinement statistics		
*R*/*R*_free_^b^	20.3/24.6	23.0/27.2
Number of atoms		
Protein	3735	3650
Solvent	26	18
RMSD		
Bond lengths, Å	0.003	0.003
Bond angles, °	0.603	0.698
Wilson *B*-factor, Å^2^	77	67
Average *B*-factor, Å^2^		
Protein	93	96
Solvent	77	71
Ligand	100	96
Ramachandran plot^c^ (residues; %)		
Preferred	441 (95.7)	429 (95.5)
Allowed	20 (4.3)	19 (4.5)
Outliers	0	0

a Values in parentheses are for the highest resolution shell.

b *R*_free_ was calculated from a subset of 5% of the data that were excluded during refinement.

c Analyzed with PROCHECK.

Importantly, the observed THC binding mode is productive and suitable for oxidation of the equally distant C7 and C8 atoms (4.1 Å from the heme iron), the preferable sites for CYP3A4-dependent metabolism (26). Because allylic hydrogens at C8 have lower bond dissociation energy than aliphatic hydrogens at C7, the C8 hydroxylation reaction would be more favorable. To allow epoxidation at the C9–C10 site (25), THC would have to flip by 180° and spatially adjust to avoid steric clashing *via* the methyl groups.

Since structural data suggest that polar contacts with S119 and R212 could help align and stabilize THC in a catalytically relevant binding mode, we tested whether the S119A and R212A mutations affect complex formation with THC. Spectral titrations showed (Fig. 1, *F* and *G*) that elimination of the polar side chain of S119 or R212 does not significantly affect the binding affinity of THC or its ability to induce high-spin transition in CYP3A4. The *K*_*d*_ value for the S119A and R212A variants was close to that for the WT: 3.0 ± 0.3 μM and 2.8 ± 0.3 μM, respectively. Thus, the long-range polar interactions with S119 and R212 assist but are not critical for the THC association, which seems to be mainly driven by hydrophobic interactions and steric complementarity with the active site.

### Interaction of CYP3A4 with CBN and CBD

**Figure 2:**
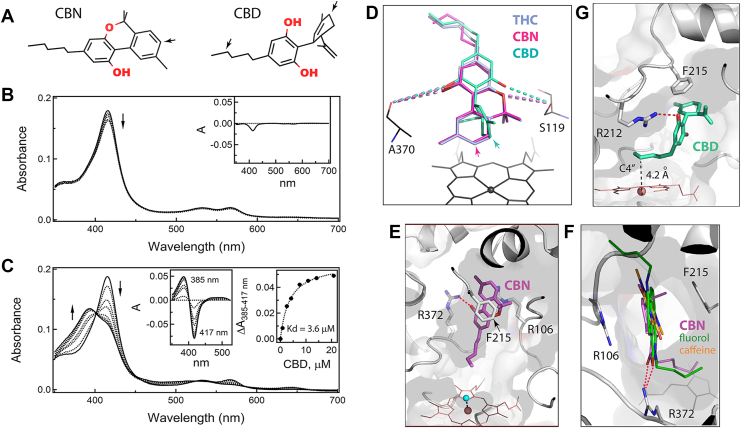
**Interaction of CYP3A4 with CBN and CBD.***A,* chemical structures of CBN and CBD in 3D projection. Sites of CYP3A4-dependent oxidation are indicated by *arrows*: C8 in CBN and C6 (major) and C4” (minor; in the aliphatic tail) in CBD. *B,* spectral changes in CYP3A4 observed during equilibrium titration with CBN. The *inset* shows a difference spectrum between the ligand-free and CBN-saturated forms (∼20 μM CBN). *C,* spectral changes in CYP3A4 observed during equilibrium titration with CBD. *Left and right insets* are the difference spectra and titration plot with hyperbolic fitting, respectively. The derived *K*_*d*_ value is indicated. Direction of absorbance changes is indicated by *arrows*. *D,* CBN and CBD manually docked at the THC binding site. Distances between the polar groups and the S119 hydroxyl and A370 carbonyl are shown as *dotted lines* and range from 4.3 to 4.6 Å. *Arrows* point at the respective metabolic sites that can be placed within 4.1–4.4 Å from the heme iron. *E,* an alternative CBN docking mode where the tricyclic moiety is stacked between the F215 ring and the R106 guanidine, and the aliphatic tail is too far from the heme to displace the distal water ligand. *F,* the proposed remote CBN-binding site coincides with the area where two other polyaromatic substrates, fluorol and caffeine, associate (8DYC, 8SO1, and 8SO2 structures). *G,* alternative reverse orientation of CBD that could promote hydroxylation at the C4” atom. This binding mode is stabilized by the stacking of the cyclohexenyl and F215 rings and H-bonding to R212. CBD, cannabidiol; CBN, cannabinol; CYP3A4, cytochrome P450 3A4.

We also investigated how CYP3A4 interacts with two other major *Cannabis sativa* constituents serving as substrates: moderately psychoactive CBN and nonpsychoactive CBD. CBN is a nonenzymatic oxidation product of THC that can be formed naturally in cannabis plants or after prolonged storage and light/heat exposure (28). CBN structurally resembles THC but has a fully aromatic C-ring (Fig. 2*A*), hydroxylated by CYP3A4 at the C8 position (25). Surprisingly, despite high structural similarity to THC, CBN causes only minor spectral changes in CYP3A4, even at saturating concentrations: a small decrease and no blue shift in the Soret band (Fig. 2*B*). This indicates that CBN alters the heme environment without displacing the axial water ligand.

CBD is less similar to THC, as it lacks the cyclic pyran structure and, instead, has an open B-ring with alkene and hydroxyl groups and a perpendicularly orientated C-ring (Fig. 2*A*). This alters the interaction of CBD with cannabinoid receptors and, as a result, profoundly decreases its psychotropic activity (29). CBD is metabolized by several hepatic CYPs, including CYP3A4, which preferably hydroxylates the C-ring C6 atom (corresponds to C8 in THC) and, to a lesser extent, C4” in the aliphatic tail (30, 31). Spectral measurements showed that, despite its distinct chemical structure, CBD induces type I spectral changes in CYP3A4 similar to those observed for THC (Fig. 2*C*) and has a comparable binding affinity (*K*_*d*_ of 3.6 ± 0.4 μM).

Both CBN and CBD resisted cocrystallization with CYP3A4. Manual ligand docking showed that these compounds could be easily superimposed with THC to place the respective sites of metabolism near the heme iron (Fig. 2*D*). Virtually, no structural adjustments were needed for CBN docking, whereas rotameric changes in R212 helped minimize steric clashing imposed by the perpendicularly oriented C-ring of CBD. In the modeled orientations, the long-range polar interactions with S119 and A370 were preserved for both compounds (4.3–4.6 Å distance between the interacting groups). Even so, according to spectral data (Fig. 2*B*), CBN does not associate in the vicinity of the heme, meaning that the higher aromaticity of the C-ring alters protein–ligand interactions and favors CBN association with a different site.

One alternative docking site identified by computer modeling (Fig. 2*E*) coincides with the area where two other substrates of CYP3A4, fluorol and caffeine, associate in the crystal structures (8DYC, 8SO1, and 8SO2; Fig. 2*F*). In this binding mode, the aliphatic tail of CBN points toward the heme, whereas the tricyclic moiety faces the substrate channel, stacking between the F215 ring and the R106 guanidine and forming an H-bond to the R372 guanidine *via* the A-ring hydroxyl. As noted previously (21, 22), the latter spot is ideally suited for the association of planar polyaromatic compounds bearing polar groups, as their binding can be stabilized through π–π, cation–π, and H-bonding interactions. For CBN, the flanking F57 and F108 provide additional hydrophobic contacts. Importantly, even in the extended conformation, the aliphatic tail of CBN is too far from the heme to displace the distal water ligand (Fig. 2*E*). Thus, binding of CBN to the intrachannel or another nearby site would preclude the high-spin transition (Fig. 2*B*) and slow down metabolism, known to be several-fold less efficient compared with THC (25).

For CBD, one possible reverse orientation that could promote C4” hydroxylation (29) is shown in Figure 2*G*, where the C4” atom is straight above and 4.2 Å from the heme iron. This binding mode is stabilized through parallel stacking of the cyclohexenyl and F215 rings and by H-bonding between the A-ring hydroxyl and the R212 guanidine. It should be noted that, for both CBN and CBD, docking in the reverse orientation required only subtle adjustments in the tilt angle of the F215 ring and/or repositioning of the R212 guanidine.

### Interaction of CYP3A4 with darifenacin

**Figure 3:**
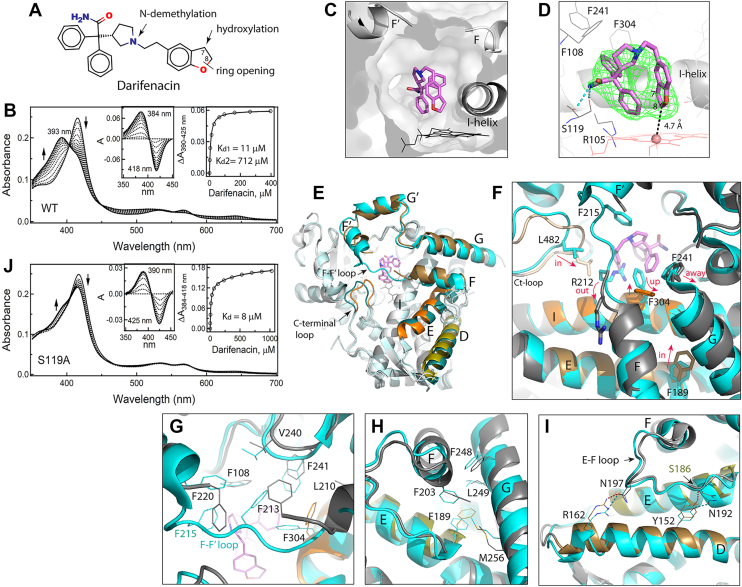
**Interaction of CYP3A4 with darifenacin.***A,* chemical structure of darifenacin. The sites of CYP3A4-dependent metabolism are indicated. *B,* spectral changes observed during equilibrium titration of WT CYP3A4 with darifenacin. Direction of absorbance changes is indicated by *arrows*. *Left and right insets* are the difference spectra and a titration plot with two-site binding fitting, respectively. The derived *K*_*d*_ values are indicated. *C,* slice through the CYP3A4 molecule with a semitransparent surface showing the orientation of darifenacin in the active site. Due to steric clashing with the F–F′ loop, residues 214 to 219 become disordered. *D,* a magnified *side view* at the active site. H-bonds between the darifenacin amide nitrogen and the S119 hydroxyl and carbonyl groups are shown as *cyan dotted lines*. *Green mesh* is a polder omit electron density map contoured at the 3.5σ level. *E,* comparison of darifenacin-bound (in *gray*) and ligand-free CYP3A4 (in various shades of *cyan*; 5VCC structure). The *F* and *G* fragment, E- and D-helices, and C-terminal loop in darifenacin-bound CYP3A4 are colored in different shades of *orange* and *olive* to highlight conformational differences with the ligand-free structure. *F,* a closer view at the active site showing conformational changes induced by darifenacin. Movements of structural elements are indicated by *arrows*. *G,* rearrangement of residues comprising the Phe-cluster. F215 and several other residues comprising the F–F’ loop are not seen in darifenacin-bound CYP3A4. *H,* structural changes in the vicinity of F189. *I,* a close-up view at the interconnected D- and E-helices and the *E* and *F* loop. H-bonds observed in ligand-free and darifenacin-bound CYP3A4 are shown as *cyan* and *red dotted lines*, respectively. *J,* spectral changes observed during equilibrium titration of CYP3A4 S119A with darifenacin. Direction of absorbance changes is indicated by *arrows*. *Left and right insets* are the difference spectra and titration plot with hyperbolic fitting, respectively. The derived *K*_*d*_ value is indicated. CYP3A4, cytochrome P450 3A4.

Darifenacin (also known as Enablex) is an M3 selective muscarinic receptor antagonist used to treat overactive bladder symptoms, like urgency, frequency, and urinary incontinence. Darifenacin has a short half-life (3–4 h) upon intravenous and immediate-release oral administration because of extensive hepatic metabolism predominantly mediated by CYP3A4 and CYP2D6 (32). The main metabolic routes are monohydroxylation in the dihydrobenzofuran ring, dihydrobenzofuran ring opening, and *N*-dealkylation of the pyrrolidine nitrogen (Fig. 3*A*) (33).

Spectral measurements showed that darifenacin is a type I ligand (Fig. 3*B*). The binding isotherm built from titration data was fitted with hyperbolic (one-site binding) and independent and cooperative two-site binding equations. Based on visual inspection and statistical metrics, the two-site binding regression model provided the best fit: the *R*^2^ value (goodness of fit) of 0.9998 *versus* 0.9869 and 0.9955 for the one-site and cooperative two-site binding models, respectively. The derived *K*_*d*_ values were 11 ± 1 μM and 712 ± 78 μM for the low- and high-affinity sites, respectively. Most of the observed absorbance change (∼70%) took place during darifenacin association to the high-affinity site. To identify this site, CYP3A4 was cocrystallized with darifenacin.

#### Crystal structure of the CYP3A4–darifenacin complex

The structure was solved to a 2.25 Ắ resolution (Table 1) and contains one darifenacin molecule in the active site. Darifenacin binds above the heme in an arched “horseshoe” conformation, folding along the ethyl linker to bring the dihydrobenzofuran close to one of the phenyl rings (3.3 Ắ apart; Fig. 3, *C* and *D*). The top of the arch clashes with the F–F′ connecting loop. As a result, R212 gets expelled from the active site, and residues 214 to 219 become disordered. The observed conformation is stabilized by two H-bonds formed between the amide nitrogen of darifenacin and the hydroxyl and carbonyl groups of S119. The phenyl moiety proximal to the heme forms cation–π interactions with the R105 guanidine, whereas another phenyl group is engaged in hydrophobic and aromatic interactions with F108, I120, F241, I301, and F304. The C8 atom of dihydrobenzofuran is the closest to and 4.7 Ắ away from the heme iron. The C7 atom, the primary hydroxylation site, is further away (5.4 Ắ) and faces the dioxygen binding groove in the I-helix. Thus, the observed crystallographic mode is productive and places darifenacin suitably for the opening and hydroxylation of the dihydrobenzofuran ring.

Comparison of darifenacin-bound and ligand-free CYP3A4 revealed how substrate-dependent changes in the active site transmit and induce large-scale alterations in the remote areas. As seen from the overlaid structures (Fig. 3*E*), besides changes in the F–F′ loop, there are notable positional shifts in the G-, E-, and D-helices and C-terminal loop. A close examination showed that darifenacin clashes not only with the F–F′ loop but also with the I-helix F304 and G-helix F241. As a result, F304 adopts a vertical rotamer, and the G-helix pulls away (Fig. 3*F*). These changes and the F215 disorder trigger reorganization in the Phe-cluster that helps optimize protein–ligand contacts (Fig. 3*G*). Further, the rotameric switch in F304 is accompanied by a small shift of the central part of the I-helix, which draws closer to darifenacin by ∼0.7 Å. This movement, as well as positional changes in the F-helix and its F203, lead to restructuring in the hydrophobic cluster centered around the E-helix F189 (Fig. 3*H*). As a result, F189 and the C-terminal end of the E-helix shift inward toward the I-helix by >2 Å. Repositioning of the E-helix and the E–F connecting loop, in turn, alters their interaction with the neighboring D-helix. The key event here is disruption of an H-bond between N192 from the E–F loop and the D-helix Y152, whose reconnection to the E-helix S186 brings the helices closer (Fig. 3*I*). Another H-bonding pair, the D-helix R162 and the E–F loop N197, follows the F-helix movements, providing an additional push for the D-helix inward shift (up to ∼1.8 Å in the C-terminal end). The largest movement, however, is observed in the C-terminal loop (residues 477–483), which shifts toward the active site by >3 Å to place the hydrophobic L482 near the dihydrobenzofuran ring of darifenacin (3.6 Å distance; Fig. 3*F*). Collectively, these conformational changes lead to contraction and rigidification of the active site, which could further decrease mobility of darifenacin and promote its oxidation.

Because the crystal structure suggests the importance of H-bonding to S119 for the complex formation with darifenacin, we checked whether the S119A mutation perturbs its binding. Spectral titrations showed (Fig. 3*J*) that elimination of the polar side chain in S119 drastically decreases the ability of darifenacin to induce high-spin transition in CYP3A4 and eliminates the low-affinity site. The fitting to the titration plot was consistent with a single-site ligand binding with *K*_*d*_ of 8 ± 1 μM. Thus, H-bonding to the S119 hydroxyl is indeed important and could stabilize the compact arched conformation of darifenacin needed for the axial water displacement. It is plausible to speculate that, in the S119A mutant, darifenacin adopts an extended conformation, which precludes association of the second molecule to the low-affinity site, likely located within the active site cavity.

### Comparison of new structures with other productive complexes with substrates

**Figure 4:**
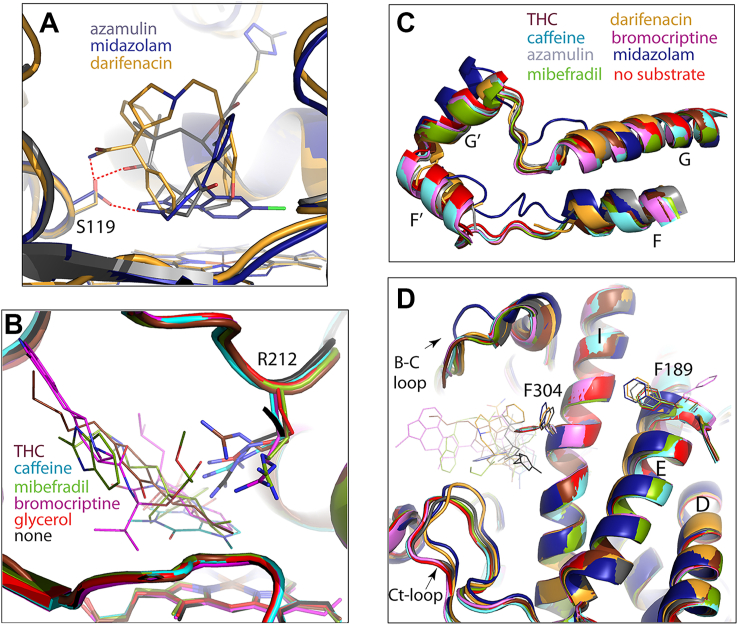
**Structural comparison of productive substrate-bound complexes of CYP3A4.***A,* besides darifenacin, azamulin and midazolam form direct H-bonds with S119 (shown in *red dotted lines*) that stabilize productive orientation. *B,* R212 adopts different conformers to optimize substrate binding through direct or water-mediated H-bonds (for caffeine, mibefradil, and bromocriptine) and/or cation–π interactions (for caffeine and THC). *C,* superposition of the F–G fragments. In most substrate-bound structures, the F–G fragment folds similarly to that in substrate-free CYP3A4. In complexes with azamulin and darifenacin, the F–F′ connector is partially disordered. In midazolam-bound CYP3A4, the entire F–G fragment undergoes reorganization. *D,* view at the central part of the protein core showing substrate-dependent differences in positioning of the F304 and F189 residues, D-, E-, and I-helices, and B–C and C-terminal loops. Protein Data Bank codes for the water-, glycerol-, azamulin-, bromocryptine-, caffeine-, midazolam- and mibefradil-bound CYP3A4 structures are 4I3Q, 5VCC, 6OOA, 3UA1, 8SO1, 5TE8 and 6OO9, respectively. CYP3A4, cytochrome P450 3A4; THC, (−)-trans-Δ9-tetrahydrocannabinol.

Polar interactions with S119 play an important role in the binding of other substrates as well. Direct H-bonding between S119 and the hydroxyl group of azamulin helps to place the pleuromutilin moiety near the heme iron (Fig. 4*A*) (15). Likewise, an H-bond with Ser119 limits movements of midazolam, a marker substrate of CYP3A4, and fixes it suitably for the C1 atom oxidation (17). Alanine at position 119 disfavors this binding mode and promotes an alternative orientation that disallows midazolam to displace the distal water ligand and favors hydroxylation at the C4 site (21).

R212 is also actively involved in substrate binding. As seen from Figure 4*B*, this flexible residue adopts various conformations to optimize substrate orientation through direct or water-mediated H-bonds (for caffeine, mibefradil, and bromocriptine) and/or cation–π interactions (for caffeine and THC). Thus, the THC- and darifenacin-bound structures reinforce the importance of S119 and R212 in creating an environment that helps to bind and metabolize chemically diverse substrates.

Comparison of the overall fold of substrate-bound complexes showed that, despite differences in chemical structures and molecular size (varying from 194 to 655 Da for caffeine and bromocriptine, respectively), most substrates do not cause conformational alterations, even in the highly flexible F–G fragment (Fig. 4*C*). This occurs because of spatial complementarity with the active site (for THC, bromocriptine, and mibefradil) or a smaller size allowing unrestricted access to the heme (for caffeine). In contrast, darifenacin, azamulin, and midazolam cause substantial structural changes. Like darifenacin, azamulin clashes with and leads to partial disorder of the F–F′ loop, whereas midazolam triggers restructuring in the entire F–G fragment (Fig. 4*C*). Another common feature is a rotameric switch in F304, which triggers rearrangement of the Phe-cluster and positional shifts in the I-, E-, and D-helices (Fig. 4*D*) through a chain of events outlined in the previous section (Fig. 3, *E*–*I*). Because the amino-triazolyl end group of azamulin overhangs over and H-bonds to the I-helix and lies in the vicinity of the C-terminal loop, conformational changes in the protein core become less pronounced. Thus, rearrangements in the F–G fragment, I/E/D-helical bundle, and C-terminal loop are substrate specific and interdependent and could be part of a mechanism that links local changes in the active site to conformational reorganization in the remote areas. This way, the F304 switch could initiate both near- and long-range structural changes that would further optimize protein–ligand contacts by altering the shape, volume, and/or rigidity of the active site. According to crystal structures, the volume of the catalytic cavity expands from 2213 Å^3^ in ligand-free CYP3A4 to 2379 Å^3^ and 3224 Å^3^ upon binding of darifenacin and azamulin, respectively, and shrinks to 1143 Å^3^ to fix small midazolam in a productive orientation.

In summary, this study provided the first mechanistic and structural insights on the interaction of CYP3A4 with cannabinoids and the drug darifenacin. The THC- and darifenacin-bound models have expanded the structural library of productive substrate-bound complexes of CYP3A4, emphasizing the importance of steric complementarity and polar interactions for ligand association and demonstrating how flexible substrates can be molded into the active site to promote site-specific oxidation. Based on structural comparison, a chain of events was identified through which substrate-dependent changes in the active site could transmit to the remote areas and alter the properties of the active site to further improve substrate binding and promote site-specific metabolism. Together, our experimental and structural results lead to better understanding of structure–function relationships and conformational dynamics of CYP3A4.

## Experimental procedures

### Materials

THC, CBD, and CBN were purchased from Sigma–Aldrich, and darifenacin was obtained from Cayman Chemical.

### Protein expression and purification

Δ3-22 truncated forms of WT, S119A, and R212A CYP3A4 were produced as reported previously (16, 34, 35).

### Spectral binding titrations

Equilibrium titrations of CYP3A4 were conducted in a Cary 300 spectrophotometer at an ambient temperature in 0.1 M phosphate, pH 7.4, containing 20% glycerol and 1 mM dithiothreitol (buffer A). Compounds were dissolved in dimethyl sulfoxide and added to a 2–2.5 μM protein solution in small aliquots, with the final solvent concentration <2%. Spectral dissociation constants (*K*_*d*_) were determined from fittings to the plots of the maximal absorbance change (peak-to-trough separations in the difference spectra; ΔA) *versus* ligand concentration. Fitting was performed using SigmaPlot software (Grafiti LLC). Equation used for one-site ligand binding:

$$Y=X/\left(K_{d}+X\right)$$

where Y represents the fraction of the occupied site, X is the total ligand concentration, and *K*_*d*_ is the equilibrium dissociation constant. Equation used for two-site ligand binding:

$$Y=\left(A_{\max\;1}\;\cdot \;X/\left(K_{d1}+X\right)\right)+\left(A_{\max\;2}\;\cdot \;X/\left(K_{d2}+X\right)\right)$$

where Y is total specific binding, X is the total ligand concentration, A_max1_ and A_max2_ are maximum absorbance changes due to specific binding to the high- and low-affinity sites, and *K*_*d*1_ and *K*_*d*2_ are the dissociation constants for the high- and low-affinity sites, respectively.

### Determination of the X-ray structures

WT CYP3A4 was cocrystallized with THC and darifenacin at room temperature by a sitting drop vapor diffusion method. Protein solution (60 mg/ml) in buffer A was mixed with a fivefold ligand excess and centrifuged to remove the precipitate. The supernatant containing ligand-bound CYP3A4 (0.4 μl) was mixed with an equal volume of the crystallization solution containing 10% PEG 3350 and 3% taximate, pH 6.0, for THC or 80 mM succinic acid, pH 7.0, for darifenacin. Crystals were harvested a few days after setup and cryoprotected with paratone-N oil before freezing in liquid nitrogen. The X-ray diffraction data were collected at the Stanford Synchrotron Radiation Lightsource beamline 12-2. Crystal structures were solved by molecular replacement with PHASER (36) and the 5VCC structure as a search model. Ligands were built with eLBOW (37) and manually fit into electron density with COOT (38). The initial models were refined with PHENIX (37) and rebuilt with COOT. Polder omit electron density maps were calculated with PHENIX. The active site volume in ligand-free and substrate-bound CYP3A4 was calculated using the CavityPlus server (http://www.pkumdl.cn:8000/cavityplus) (39). Data collection and refinement statistics are summarized in Table 1. The atomic coordinates and structure factors for the THC- and darifenacin-bound CYP3A4 were deposited to the Protein Data Bank with the ID codes 9PLJ and 9PLK, respectively.

## Data availability

All experimental data generated during this study are included in this article. Coordinates and structure factors for the X-ray models of THC- and darifenacin-bound CYP3A4 are freely available at the Protein Data Bank (https://www.rcsb.org/).

## Conflict of interest

The authors declare that they have no conflicts of interest with the contents of this article.
